# Environmentally-Coupled Signs and Gestures

**DOI:** 10.5334/joc.132

**Published:** 2021-08-23

**Authors:** Karen Emmorey

**Affiliations:** 1 San Diego State University, San Diego, US

**Keywords:** Embodied cognition, Language production, Semantics

## Abstract

Environmentally-coupled gestures are defined by Goodwin (2007) as gestures that can only be interpreted by taking into account the physical environment of the speaker. Lexical signs, unlike spoken words, can be also be environmentally-coupled because the visual-manual modality allows for signs to be articulated on or near elements in the environment. The speech articulators are largely hidden from view and do not permit environmental coupling. This commentary provides examples of environmentally-coupled signs, which can only be explained within a language-as-situated approach. However, such expressions are also constrained by internal, systematic properties of language, indicating that both language-as-situated and language-as-system approaches are necessary to account for the non-arbitrary (iconic and indexical) properties of language.

Chuck Goodwin (2007) identified environmentally-coupled gestures as gestures that cannot be understood without taking into account the environment to which they are tied. Deictic gestures are a primary example, e.g., one can only interpret what is meant by a point accompanying the exclamation “I want that!” by linking the gesture to an object in the environment. Gestures are often grounded in the physical environment because people gesture toward objects, events, or locations that they are talking about. These types of multi-modal expressions can only be accounted for under the language-as-situated view promoted by Murgiano, Motamedi, and Vigliocco (2020).

Here I propose that signers can exploit unique properties of the visual-manual modality to create *environmentally-coupled signs* and that this phenomenon is not possible for spoken words. That is, lexical signs, unlike words, can be coupled with the environment. An illustration of this phenomenon was described in Emmorey (2001). In these examples, a deaf teacher produced lexical (non-pointing) signs on the chalkboard during her explanation in American Sign Language (ASL) of physical and chemical change to her class of deaf third-grade students. The teacher had written the words “gas,” “liquid,” and “solid,” vertically on the board, in top to bottom order. She pointed to the word “gas” and asked a child, “what happens when it gets cold?” When the child answered incorrectly, she signed NO #BACK, producing the fingerspelled loan sign #BACK from the word “gas” to the word “liquid” on the chalkboard. Next to these three words, she had written the physical examples “steam,” “water,” and “ice.” She pointed to each word and then signed THREE #ALL LOOK-SAME? SAME? She produced the signs #ALL and SAME (Y handshape) vertically over the three words, and by doing so, she grouped together these words and concepts. A third example of environmentally-coupled signing was a sign tied to a drawing of the sun at the top corner of the chalkboard, with the English word “evaporation” written underneath. The teacher articulated the ASL sign EVAPORATE, which iconically depicts upward absorption, directly on the chalkboard toward both the sun drawing and the English word. In this way, the teacher linked the ASL sign to the English translation and illustrated the evaporation process as well.

Although both speakers and signers can couple deictic gestures/signs with the environment, the ability to physically direct lexical forms toward the environment appears to be uniquely available to signed languages. Speakers can gesture with some of their vocal articulators, as evidenced by the use of lip-pointing; however, such lip gestures occur in structured combination with speech, rather than articulated simultaneously with a spoken word (Enfield, 2001). Because the speech articulators are largely hidden from view while talking, it is not possible to articulate a spoken word on or near an element in the environment. In contrast, lexical signs that are produced in neutral space (i.e., are not body-anchored) can be directed toward and coupled with elements in the environment. Such expressions can only be interpreted from a language-as-situated perspective.

Nonetheless, the language-as-system account is also at play in constraining and interpreting environmentally-coupled lexical signs. I suggest that the Double Mapping Constraint (DMC) on metaphorical extensions proposed by Meir (2010) applies to environmentally-coupled signs. For example, the ASL sign MELT has a downward motion that depicts the visual perception of the height of a solid decreasing as it melts. It would be decidedly odd for the teacher to produce MELT from the word “solid” to “liquid” as they were written on the chalkboard. Although the movement would accurately express the change of state from a solid to a liquid, the upward direction of this environmental-coupling would conflict with the iconic mapping of the sign and thus violate the DMC. Further investigations of this phenomenon are likely to reveal other language-internal (i.e., phonological, morphological, or syntactic) constraints on how, when, and what types of lexical signs can be coupled with the environment.

In sum, both the language-as-system and language-as-situated approaches are necessary for a full account of how non-arbitrariness (iconicity and indexicality) are deployed in human language.

## References

[1] Emmorey, K. (2001). Space on hand: The exploitation of signing space to illustrate abstract thought. In M. Gattis (Ed.), Spatial schemas and abstract thought (pp. 147–174).Cambridge, MA: The MIT Press.

[2] Enfield, N. J. (2001). ‘Lip-pointing’: A discussion of form and function with reference to data from Laos. Gesture, 1(2), 185–211. DOI: 10.1075/gest.1.2.06enf

[3] Goodwin, C. (2007). Environmentally coupled gestures. In S. Duncan, J. Cassell & E. Levy (Eds.), Gesture and the dynamic dimension of language (pp. 195–212). Amsterdam, The Netherlands: John Benjamins. DOI: 10.1075/gs.1.18goo

[4] Meir, I. (2010). Iconicity and metaphor: Constraints on metaphorical extension of iconic forms. Language, 86(4), 865–896. DOI: 10.1353/lan.2010.0044

[5] Murgiano, M., Motamedi, Y., & Vigliocco, G. (2020). Situating language in the real-world: the role of multimodal iconicity and indexicality. Journal of Cognition, 3(1), 38. DOI: 10.5334/joc.113 34514309 PMC8396123

